# Supplementary material for: An innate granuloma eradicates an environmental pathogen using Gsdmd and Nos2
Source: Nat Commun. 2023 Oct 21;14:6686. doi: 10.1038/s41467-023-42218-1 (PMC10590453; doi:10.1038/s41467-023-42218-1)
Supplement: Supplementary file 1 — Supplementary Information [file 41467_2023_42218_MOESM1_ESM.pdf]

Supplementary Information for

An innate granuloma eradicates an environmental pathogen using *Gsdmd* and *Nos2*

Carissa K. Harvest[1,2,3], Taylor J. Abele[1,2], Chen Yu[4], Cole J. Beatty[1,4], Megan E. Amason[1,2,3], Zachary P. Billman[1,2,3], Morgan A. DePrizio[1,2], Fernando W. Souza[1,2,5], Carolyn A. Lacey[1,2], Vivien I. Maltez[3], Heather N. Larson[1,2], Benjamin D. McGlaughon[3], Daniel R. Saban[1,4], Stephanie A. Montgomery[6], and Edward A. Miao[1,2,5,7]

[1] Department of Integrative Immunobiology, Duke University School of Medicine, Durham, NC, USA
[2] Department of Molecular Genetics and Microbiology, Duke University School of Medicine, Durham, NC, USA
[3] Department of Microbiology and Immunology, University of North Carolina at Chapel Hill, Chapel Hill, NC, USA
[4] Department of Ophthalmology, Duke University School of Medicine, Durham, NC, USA
[5] Department of Cell Biology, Duke University School of Medicine, Durham, NC, USA
[6] Department of Pathology, University of North Carolina at Chapel Hill, Chapel Hill, NC, USA
[7] Department of Pathology, Duke University School of Medicine, Durham, NC, USA

Correspondence to:
Edward A. Miao
edward.miao@duke.edu
919-668-7555

Supplementary Figures

Supplementary Figure 1.

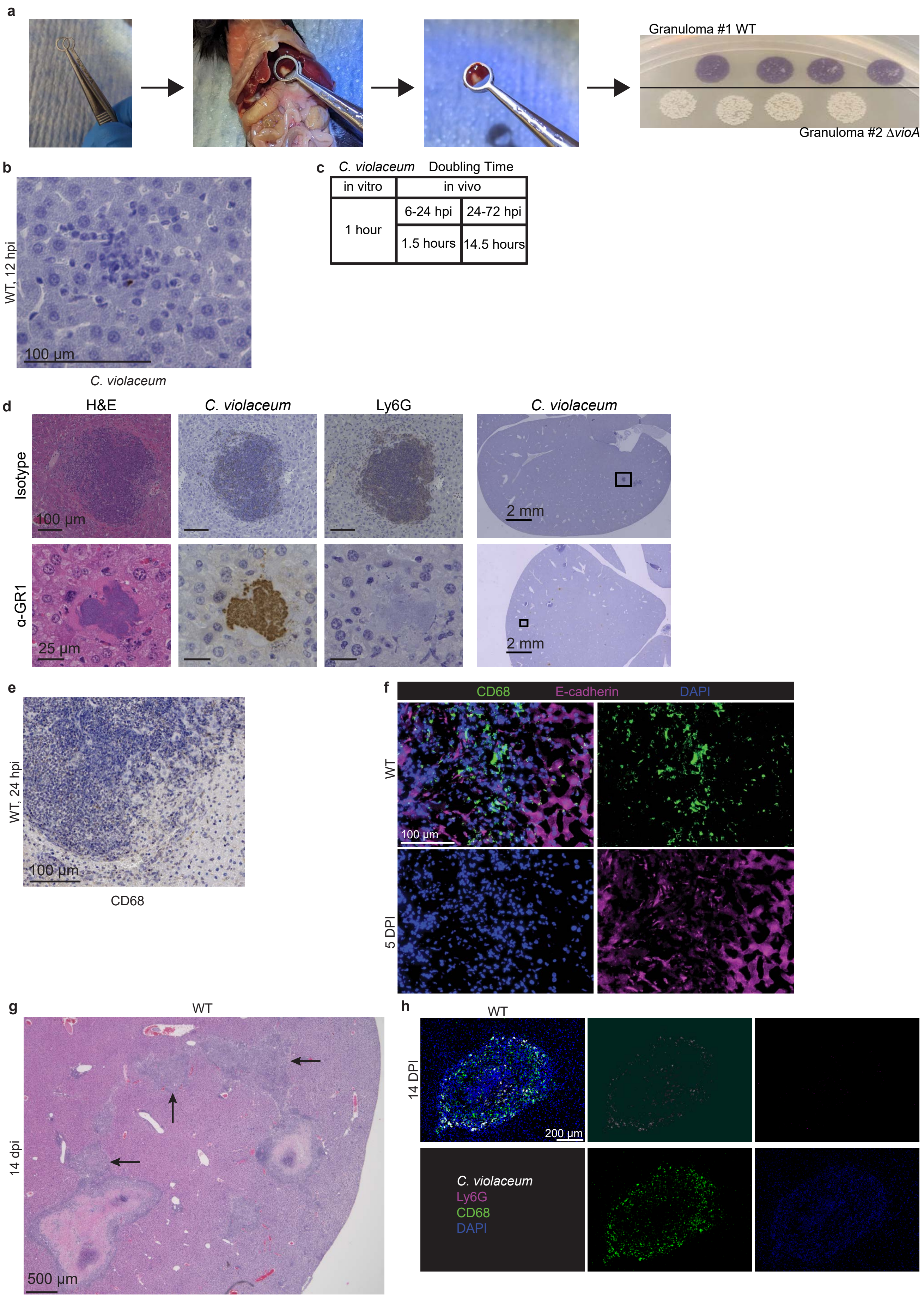

Supplemental Figure 1. Characterization of the *C. violaceum*-induced granuloma.
(a-g) Mice were infected with 10^4 CFU WT *C. violaceum*, strains indicated.
(a) Inoculum was a 1:1 mixture of WT and Δ*vioA* *C. violaceum*. Schematic for harvesting single granulomas using circular tipped tweezers. BHI agar plate representative of two, single granulomas plated CFUs 5 dpi.
(b) Visualization of *C. violaceum* within liver Kupffer cells and infiltrating immune cells 12 hpi. Representative of 2 experiments, each with 3-4 mice.
(c) *C. violaceum* replication rates in vitro in BHI broth and in vivo via liver CFUs, displayed as doubling time. In vitro representative of 2 experiments. Data used from Figure 1j for 6-24 hpi in vivo and from Figure S2a for 24-72 hpi in vivo.
(d) Mice were treated with either anti-GR1 or isotype control at day -1 and 0, then infected on day 0 with *C. violaceum*. Mice were harvested at 24 hpi for H&E staining or for the indicated IHC. The *C. violaceum* stained IHC images shown in Figure 1h are from the same experiment.
(e) Zoomed in CD68 staining of WT 1 dpi lesion from Figure 1j.
(f) Zoomed in IF staining of WT 5 dpi granuloma with indicated markers. Representative of 2 experiments, each with 3-4 mice, each with multiple granulomas per section.
(g) H&E staining of WT resolving granulomas with no necrotic core 14 dpi from Figure 3a.
(h) WT liver sections stained with indicated IF markers 14 dpi. Representative of 2 experiments, each with 4 mice, each with multiple granulomas per section.

Supplementary Figure 2.

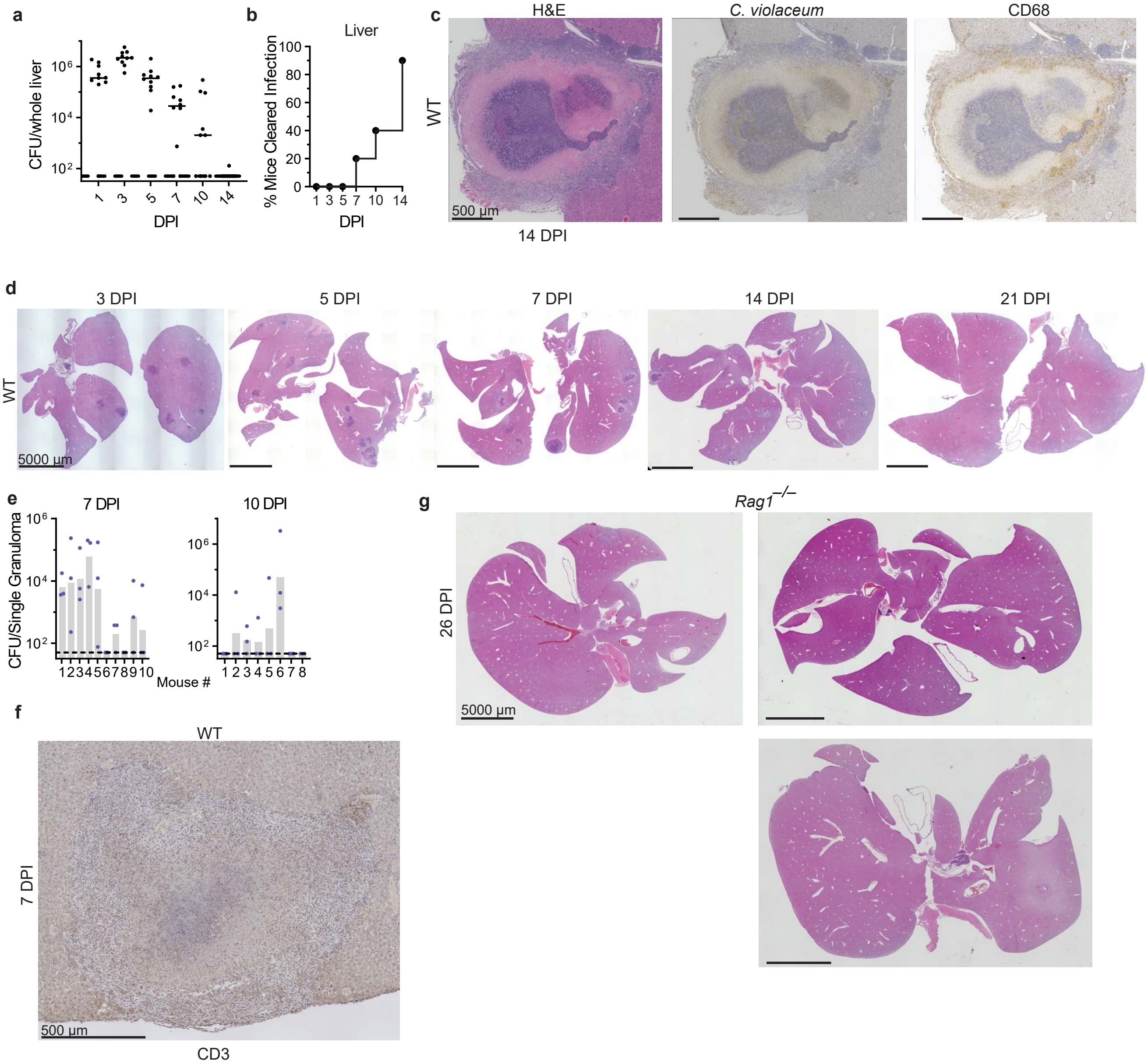

Supplemental Figure 2. Kinetics of granuloma resolution.
(a-g) Mice were infected with 10^4 CFU WT *C. violaceum.*
(a) Bacterial burdens in the liver at indicated dpi. Data combined from 2 experiments; each point is a single mouse.
(b) Bacterial burdens from (a) displayed as % bacterial clearance.
(c) Serial sections of WT liver stained by H&E or indicated IHC markers of large granuloma from Figure 3a.
(d) Representative WT whole liver sections stained with H&E, used for percent active inflammation quantification from Figure 3g. Whole livers for 5, 7, 14, 21 dpi are same as whole livers in Figures 2a, 3a, and 3c.
(e) Bacterial burdens from single WT granulomas 7 and 10 dpi. Representative of 2 experiments; each point is a single granuloma; each letter is a mouse.
(f) WT granuloma stained for CD3 7 dpi. Representative of 5 experiments.
(g) H&E staining of *Rag1*$^{-/-}$ livers at 26 dpi. Data represents 1 experiment with 3 mice.

Supplementary Figure 3.

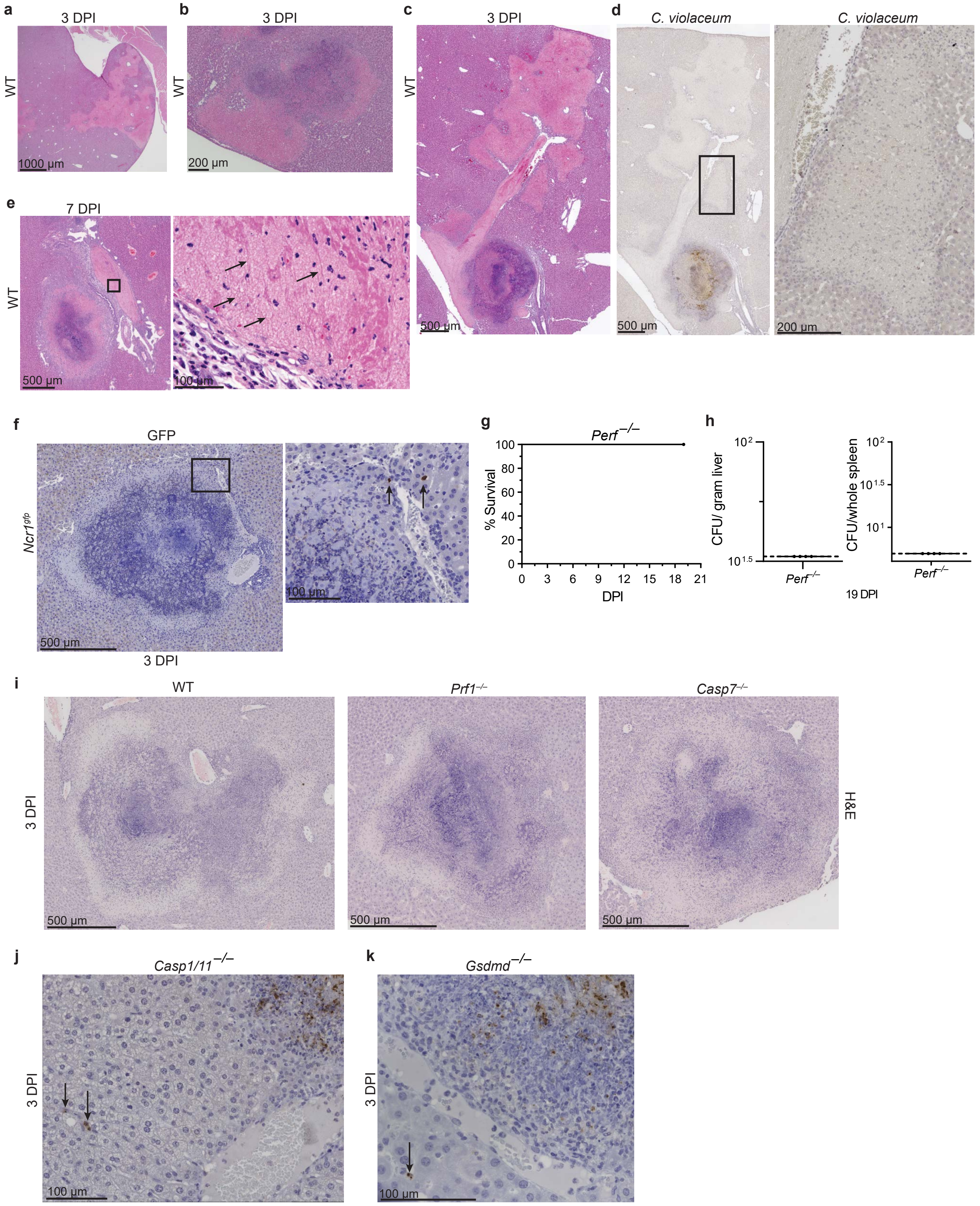

Supplemental Figure 3. Thrombosis, ischemia, and other aspects of the granuloma.
(a-k) Mice were infected with 10^4 CFU WT *C. violaceum.* Dashed line, limit of detection; solid line, median.
(a) Isolated ischemia stained with H&E 3 dpi. Representative of 10 experiments, each with 3-4 mice, each with multiple areas per section.
(b) Adjacent ischemia stained with H&E 3 dpi. Representative of 10 experiments, each with 3-4 mice, each with multiple areas per section.
(c and d) Adjacent ischemia stained with H&E or IHC for *C. violaceum* 3 dpi. Representative of 10 experiments, each with 3-4 mice, each with multiple areas per section.
(e) Clot stained with H&E 7 dpi. Arrows, fibrin strands. Representative of 5 experiments, each with 3-4 mice, each with multiple clots per section.
(f) *Ncr1*^{gfp} liver stained for indicated IHC marker 3 dpi. Arrows, NK cells. Data represents1 experiment with 3 mice.
(g) Survival analysis of *Perf*$^{-/-}$ mice. Data represents 1 experiment with 4 mice.
(h) Bacterial burdens from liver and spleen 19 dpi from (g); each point is a single mouse.
(i) Representative granuloma histology of WT, *Perf*$^{-/-}$, and *Casp7*$^{-/-}$ mice 3 dpi stained for H&E. Histopathology analyses were performed on livers from 4 mice per time point from 1 experiment.
(j) IHC staining of *C. violaceum* dissemination in liver of *Casp1*$^{-/-}$*Casp11*$^{-/-}$ (*Casp1/11*$^{-/-}$) mice 3 dpi. Representative of 2 experiments. Arrows, disseminated bacteria.
(k) IHC staining of *C. violaceum* dissemination in liver of *Gsdmd*$^{-/-}$ mice 3 dpi. Representative of 2 experiments. Arrows, disseminated bacteria.

Supplementary Figure 4.

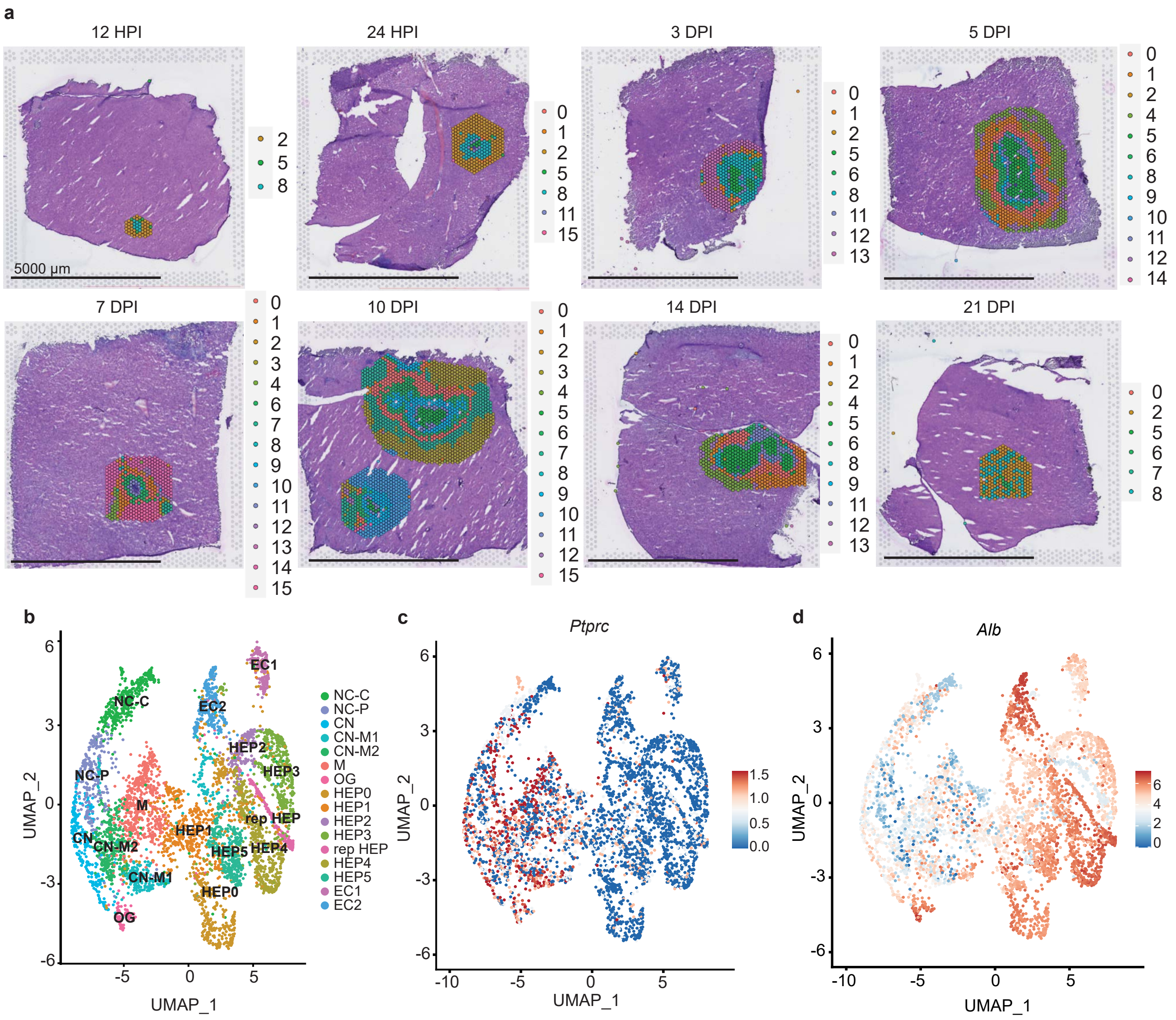

e

Cell Type	Marker Genes Used for Modules
Neutrophil	*S100a8, S100a9, Cd33, Csf3r, Ccl3*
T Cells	*Cd3d, Cd3e, Cd3g, Trac*
Monocytes	*Ly6c2, Ccr2, Fcgr3, Cd14, Itgam*
Macrophages	*Aif1, Adgre1, Fcgr1, Cd68, C1qa, C1qb, C1qc*
Fibroblasts	*Col1a1, Col1a2, Col3a1, Col5a1, Col5a2*
Hepatocytes	*Fabp1, Mup20, Apoa2, Apoc1, Mup3, Apoc3, Scd1, Rbp4, Serpina3k, Alb*
Endothelial Cells	*Clec4g, Kdr, Aqp1, Ptprb, Fabp4*

Supplemental Figure 4. Spatial transcriptomics of the granuloma.
(a-f) Mice were infected with 10^4 CFU *C. violaceum* and one liver was harvested per indicated timepoint for spatial transcriptomics analysis.
(a) H&E and spatial transcriptomic orientation of indicated clusters from all timepoints; same granuloma at 5 dpi from Figure 5a.
(b) UMAP plot for cluster expression orientation for clusters' location from all timepoints of infection within the granuloma architecture. Necrotic core (NC), necrotic core center (NC-C), necrotic core periphery (NC-P), coagulative necrotic zone (CN), CN-macrophage zone (CN-M), macrophage zone (M), outside granuloma (OG), hepatocyte (HEP), and endothelial cell (EC).
(c) UMAP plot for cluster expression orientation for clusters from all timepoints of infection expressing *Ptprc*.
(d) UMAP plot for cluster expression orientation for clusters from all timepoints of infection expressing *Alb*.
(e) Table of genes used for calculating module scores in Figure 5d.

Supplementary Figure 5.

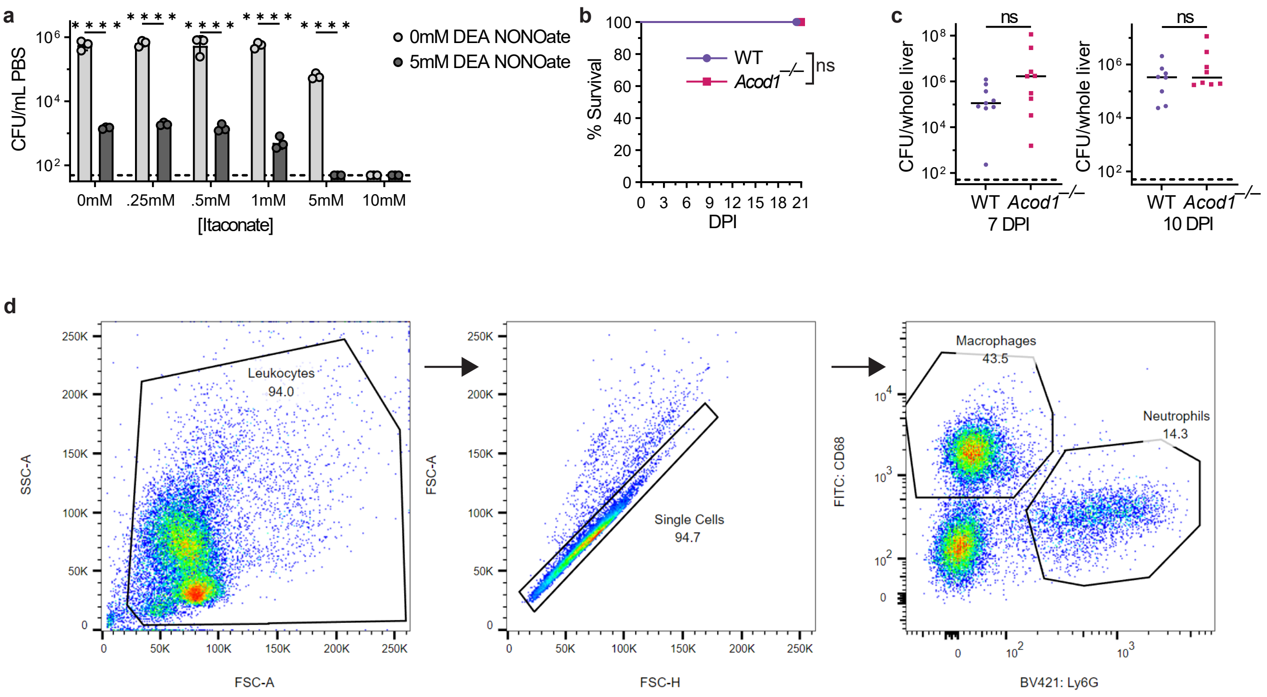

Supplemental Figure 5. Analysis of the role of nitric oxide or itaconate during *C. violaceum* infection.

(a-d) Mice were infected with 10^4 CFU WT *C. violaceum.* Dashed line, limit of detection; solid line, median.

(a) Bacterial burdens from *C. violaceum* incubated at indicated concentrations of DEA NONOate and Itaconic acid after 3 hours. Representative of 2 experiments; each point is a single culture. ****p<.0001, by Two-way ANOVA. Bar represents mean ± SD.

(b) Survival analysis of WT and *Acod1*$^{-/-}$ mice. N= 5 (WT) or 4 (*Acod1*$^{-/-}$) mice. Representative of 2 experiments, each with 4-5 mice per genotype. ns, not significant, by Kaplan-Meier survival analysis.

(c) Bacterial burdens from liver 7 and 10 dpi. Data combined from 2 experiments; each point is a single mouse. ns, not significant, by Mann-Whitney (7 dpi) and Unpaired two-tailed t test (10 dpi).

(d) Gating strategy for Figure 6e.

Supplementary Figure 6.

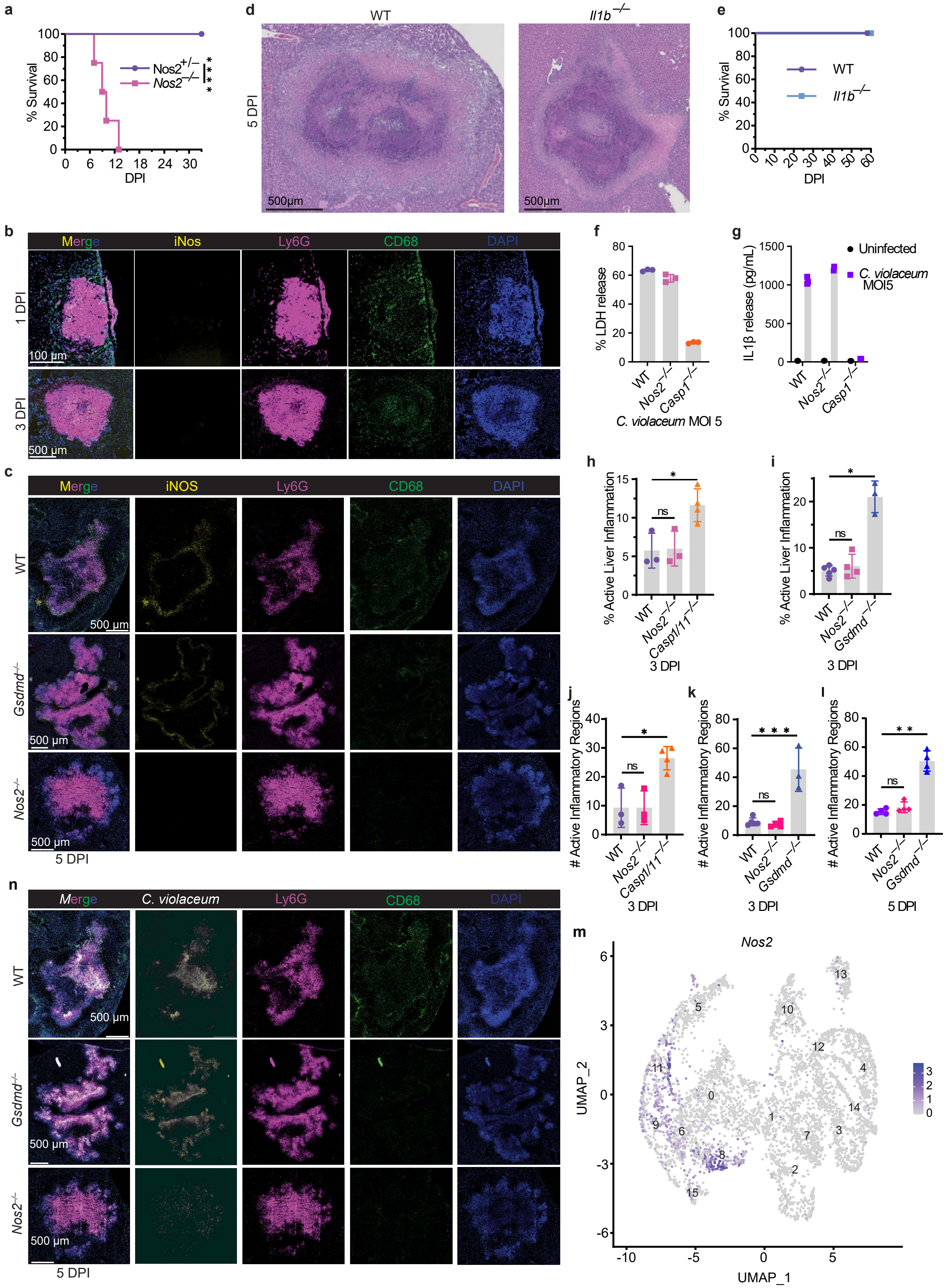

Supplemental Figure 6. Analysis of the role of gasdermin D and iNOS in the granuloma.
(a-n) Mice were infected with 10^4 CFU WT *C. violaceum.*
(a) Survival analysis of *Nos2*$^{-/-}$ and littermate *Nos2*$^{+/-}$ mice. Data represents1 experiment with 4 *Nos2*$^{-/-}$ and 11 *Nos2*$^{+/-}$ mice. ****p<.0001, by Kaplan-Meier survival analysis.
(b) WT liver sections stained with indicated IF markers 1 and 3 dpi. Representative of 2 experiments, each with 4 mice, each with multiple granulomas per section.
(c) WT, *Gsdmd*$^{-/-}$, and *Nos2*$^{-/-}$ liver sections stained with indicated IF markers 5 dpi. Same WT and *Gsdmd*$^{-/-}$granulomas as Figure 7h. Representative of 2 experiments, each with 3-4 mice per genotype, each with multiple granulomas per section.
(d) Representative granuloma histology of WT and *Il1b*$^{-/-}$ mice at 5 dpi stained for H&E. Histopathology analyses were performed on livers from 4 mice per timepoint from 1 experiment.
(e) Survival analysis of WT and *Il1b*$^{-/-}$ mice. Data represents 1 experiment with 6 WT mice and 5 *Il1b*$^{-/-}$ mice.
(f) LDH release from bone marrow derived macrophages infected with MOI 5 *C. violaceum* for 1 hour. Data are represented as mean ± SD of 3 technical replicates from 1 experiment.
(g) IL1β release from bone marrow derived macrophages infected with MOI 5 *C. violaceum* for 1 hour. Data are represented as mean ± SD of 3 technical replicates from 1 experiment.
(h,j) Percent active inflammation (h) or number of active inflammatory regions (j) quantified per whole liver section stained with H&E 3 dpi. n= 3 (WT, *Nos2*$^{-/-}$) or 4 (*Casp1/11*$^{-/-}$) mice. Representative of 2 experiments, each with 3-4 mice per genotype; each point is a single mouse. n.s, not significant, *p=0.0201 (h) or *p=0.0111 (j), by One-way ANOVA. Bars represent mean ± SD.
(i,k) Percent active inflammation (i) or number of active inflammatory regions (k) quantified per whole liver section stained with H&E 3 dpi. n= 5 (WT), 4 (*Nos2*$^{-/-}$) or 3 (*Gsdmd*$^{-/-}$) mice. Representative of 2 experiments, each with 3-4 mice per genotype; each point is a single mouse. n.s, not significant, *p=0.0301 (i) or ***p=0.0002 (k), by One-way ANOVA. Bars represent mean ± SD.
(l) Number of active inflammatory regions quantified per whole liver section stained with H&E 5 dpi from Figure 7d and 7e. Representative of 2 experiments, each with 4 mice per genotype; each point is a single mouse. n.s, not significant, **p=.0056, by One-way ANOVA. Bars represent mean ± SD.
(m) UMAP plot for *Nos2* expression orientation for clusters from all timepoints of infection.
(n) WT, *Gsdmd*$^{-/-}$, and *Nos2*$^{-/-}$ liver sections stained with indicated IF markers 5 dpi. Same WT and *Gsdmd*$^{-/-}$granulomas as Figure 7h. Representative of 2 experiments, each with 3-4 mice per genotype, each with multiple granulomas per section.

Supplementary Methods

Calculating *C. violaceum* Replication Rate

C. violaceum was grown overnight as previously described. *C. violaceum* was then back-diluted at 1:100 in 20 mL BHI broth. 1 mL samples were taken every hour to measure OD. These data were used to calculate in vitro doubling time. For in vivo doubling time, CFUs from whole liver samples were taken every 6 hpi for the first 24 hours of infection, or every 24 hours up to 72 hpi. Doubling time was determined by comparison to a set of theoretical doubling CFUs based on the data collected at either 6 or 24 hpi.

In vitro Killing of *C. violaceum* with Itaconate and Nitric Oxide

C. violaceum was grown overnight in BHI broth then diluted to 1 x 10^6 CFU/mL in PBS and mixed with various concentrations of DEA NONOate (Cayman Chemical Cat. No. 82100) with or without itaconic acid (Sigma-Aldrich Cat. No. I29204). Cultures were incubated at room temperature for 3 hours then diluted at 1:5 in sterile PBS and plated on BHI agar to assess bacterial viability.

Cytotoxicity Assay and IL-1β ELISA

Primary bone marrow macrophages were isolated as described[40]. Media consisted of DMEM (Thermo Fisher Cat. 11995073) with 10% FBS and 15% L-Cell conditioned Media. Penicillin and streptomycin were added to the media during differentiation and thawing but withheld during seeding and infection. Macrophages were seeded at 4x10^4 cells/well in 96-well TC treated plates and infected with MOI 5 *C. violaceum*, grown overnight at 37°C shaking. Infected cells were then centrifuged for 5 minutes at 200g, incubated for 1 hour, then supernatants were collected for assay and frozen at -80°C until analysis. Lipopolysaccharide (50ng/mL) was added to normalize TLR activation for mock infected cells. Cytotoxicity was determined by lactate dehydrogenase assay (CytoTox 96, Promega, Cat. G1780). IL-1β release was determined via ELISA (Mouse IL-1 beta/IL-1F2 DuoSet ELISA, R&D Systems, Cat. DY401). Samples were read on a BioTek Synergy H1 microplate reader running Gen5 ver 3.10 software. All samples and reagents were brought to room temperature before analyzing. 3 technical replicates performed per condition, data from 1 independent experiment.

Supplementary Notes

Ischemia and coagulation outside the granuloma

We observed other pathology outside the granuloma structure. Ischemia was observed in WT mice at 3 and 5 dpi (Figure 2a and S3a), often directly adjacent to a granuloma (Figure S3b and S3c), suggesting that the granuloma occluded or affected the blood supply. Ischemia observed in isolation could be adjacent to a granuloma outside the histologic plane. Ischemic areas were of various sizes and did not stain for *C. violaceum* antigens (Figure S3d). We did not observe ischemia past 7 dpi suggesting rapid regeneration, consistent with the robust regenerative power of hepatocytes. We frequently observed thrombosis in the liver vasculature, some of which occluded hepatic vessels and caused ischemia in the downstream tissue (Figure S3c and S3e).

Supplementary References

1.Goncalves, A.V., Margolis, S.R., Quirino, G.F.S., Mascarenhas, D.P.A., Rauch, I., Nichols, R.D., Ansaldo, E., Fontana, M.F., Vance, R.E., and Zamboni, D.S. (2019). Gasdermin-D and Caspase-7 are the key Caspase-1/8 substrates downstream of the NAIP5/NLRC4 inflammasome required for restriction of Legionella pneumophila. PLoS Pathog *15*, e100788610.1371/journal.ppat.1007886.
