# Supplementary material for: An innate granuloma eradicates an environmental pathogen using Gsdmd and Nos2
Source: Nat Commun. 2023 Oct 21;14:6686. doi: 10.1038/s41467-023-42218-1 (PMC10590453; doi:10.1038/s41467-023-42218-1)
Supplement: Supplementary file 3 — Description of Additional Supplementary Files [file 41467_2023_42218_MOESM3_ESM.pdf]

Description of Additional Supplementary Files

File Name: Supplementary Data 1
Description: Raw dataset generated from spatial transcriptomics.

File Name: Supplementary Data 2
Description: Code used for analyzing spatial transcriptomic data.
