# Supplementary material for: An innate granuloma eradicates an environmental pathogen using Gsdmd and Nos2
Source: Nat Commun. 2023 Oct 21;14:6686. doi: 10.1038/s41467-023-42218-1 (PMC10590453; doi:10.1038/s41467-023-42218-1)
Supplement: Supplementary file 5 — Supplementary Data 2 [file 41467_2023_42218_MOESM5_ESM.pdf]

Spatial Transcriptomics data analysis using R codes:

```
library(Seurat)
library(tidyverse)
library(showtext)
library(scales)
library(RColorBrewer)

#Fig 5a – Spatial Feature Plots of Clusters
Liver_Spatial <- readRDS("/Users/eam119/Documents/RStudio/mergered8_clustree.rds")
Idents(Liver_Spatial) <- Liver_Spatial$SCT_snn_res.0.9
mylist <- hue_pal()(16)
Liver_Spatial <- RunPCA(Liver_Spatial, assay = "SCT", verbose = FALSE)
Liver_Spatial <- FindNeighbors(Liver_Spatial, reduction = "pca", dims = 1:30)
Liver_Spatial <- FindClusters(Liver_Spatial, verbose = FALSE)
Liver_Spatial <- RunUMAP(Liver_Spatial, reduction = "pca", dims = 1:30)
DimPlot(Liver_Spatial, reduction = "umap", label = TRUE)


For #Fig 5b-e
font_add(family = "Arial", regular = "Arial.ttf") #Prepare Arial font for use in figures
showtext_auto() #Tell system to use showtext to generate fonts by default

#Load dataset and color palette
df <- readRDS("~/Desktop/mergered8_clustree.rds")
Idents(df) <- df$SCT_snn_res.0.9
mylist <- hue_pal()(16)

#[1] "#F8766D" "#E68613" "#CD9600" "#ABA300" "#7CAE00" "#0CB702" "#00BE67" "#00C19A"
#"#00BFC4" "#00B8E7" "#00A9FF" "#8494FF" "#C77CFF" "#ED68ED" "#FF61CC" "#FF68A1"

#Fig 5b and S4b - DimPlot with *numbered clusters* and Arial font for all text and points
DimPlot(df) %>% LabelClusters(id = "ident", repel = F) +
theme(text=element_text(family="Arial")) + NoLegend()

#Fig 5c, S4c, and S4d - Dimplot with grayed-out Ptprc low/- clusters
levels(df) <- c("5","11","9","8","6","0","15","2","1","12","4","14","3","7","13","10") #Reorder
clusters based on physical location in the granuloma - approximately center of granuloma to
edge
df <- RenameIdents(df, "5" = "NC-C","11" = "NC-P","9" = "CN","8" = "CN-M1", "6"= "CN-M2","0"
= "M",
        "15" = "OG","2" = "HEP", "1"="HEP", "12"= "HEP","4"= "HEP","14"= "HEP","3"=
"HEP","7"= "HEP",
```

```
                "13" = "EC","10" = "EC") #Renaming clusters based on physical location in the
granuloma and gene expression
df[["zones"]] <- Idents(df) #Save new labels as "zones"
#FeaturePlot(df, c("Ptprc"), max.cutoff = 1.5) + scale_colour_gradientn(colours =
rev(brewer.pal(n = 7, name = "RdBu"))) + theme(text=element_text(family="Arial")) #Check
Ptprc expression to confirm cluster selections
DimPlot(df, cols = c(mylist[6],mylist[12],mylist[10],mylist[9],mylist[7],mylist[1],
                        mylist[16],"grey","grey")) %>% LabelClusters(id = "ident", family = "Arial",
repel = F) + theme(text=element_text(family="Arial"))

#Fig 5d - Modules Over Real Time Violin Plot - All Spots
### create gene module lists
pmn.list <- list(c("S100a8","S100a9","Cd33","Csf3r","Ccl3"))
mo.list <- list(c("Ly6c2","Ccr2","Fcgr3","Cd14","Itgam"))
mac.list <- list(c("Aif1","Adgre1","Fcgr1","Cd68","C1qa","C1qb","C1qc"))
t.list <- list(c("Cd3d","Cd3e","Cd3g","Trac"))
fibro.list <- list(c("Col1a1","Col1a2","Col3a1","Col5a1","Col5a2"))
hep.list <-
list(c("Fabp1","Mup20","Apoa2","Apoc1","Mup3","Apoc3","Scd1","Rbp4","Serpina3k","Alb"))
ec.list <- list(c("Clec4g","Kdr","Aqp1","Ptprb","Fabp4"))
### add gene module scores
df <- AddModuleScore(df, features = pmn.list, name = "PMN_score")
df <- AddModuleScore(df, features = t.list, name = "T_score")
df <- AddModuleScore(df, features = mo.list, name = "MO_score")
df <- AddModuleScore(df, features = mac.list, name = "MAC_score")
df <- AddModuleScore(df, features = fibro.list, name = "FIBRO_score")
df <- AddModuleScore(df, features = hep.list, name = "HEP_score")
df <- AddModuleScore(df, features = ec.list, name = "EC_score")

df$library <- factor(df$library, c("1_12_hpi", "7_24_hpi", "9_3_dpi", "10_5_dpi", "11_7_dpi",
"12_10dpi", "13_14dpi", "14_21dpi")) #Reordering the timepoints
Timepoints <- c("0.5dpi", "1dpi", "3dpi", "5dpi", "7dpi", "10dpi", "14dpi", "21dpi") #New names
for the timepoints
levels(df$library) <- Timepoints #Renaming timepoints using the list generated
Idents(df) <- factor(df$library) #Setting Timepoints as Idents
colnames(df@meta.data)[21:27] <- c("Neutrophil", "T Cell", "Monocyte", "Macrophage",
"Fibroblast", "Hepatocyte", "Endothelial Cell") #Rename module scores to associated cell
annotation
VlnPlot(df, c("Neutrophil","Monocyte", "Macrophage","T Cell", "Fibroblast"),
    ncol = 5, pt.size = 0) &
  ylim(-2,4) &
  theme(text=element_text(family="Arial"))

#Fig 5e - Module Analysis Dot Plot
```

```
Idents(df) <- df$zones #Change idents to physical zone labels
p0 <- DotPlot(df,features = c("Neutrophil","Monocyte", "Macrophage","T Cell", "Fibroblast",
"Hepatocyte", "Endothelial Cell"),scale = T,dot.scale = 6) #Create and store dotplot based on
module scores of each zone in respect to each cell type
p0 + scale_colour_gradientn(colours = (brewer.pal(n = 7, name = "YlOrRd"))) + #Update visuals
of dotplot
  theme_grey(base_size = 14) +
  RotatedAxis() +
  scale_y_discrete(limits=rev) + xlab("Module") +
  ylab("Zone") +
  guides(size = guide_legend(title.position="left", title.hjust = 0.5, guide_legend(title = "%
Exp.")), colour = guide_colourbar(title.position="left", title.hjust = 1, title = "Average Exp.")) +
  theme(legend.title = element_text(angle = 90)) +
  theme(text=element_text(family="Arial"))

#Fig 5f, 6f, and S4a – Spatial Feature Plots of indicated genes
SpatialDimPlot(Liver_Spatial)& NoLegend()
SpatialFeaturePlot(Liver_Spatial, "Trem2", crop = F, ncol = 4) & scale_fill_gradientn(colours =
rev(brewer.pal(n = 11, name = "RdYlBu")), limits = c(0,3), oob=squish)
```
