# Supplementary material for: An innate granuloma eradicates an environmental pathogen using Gsdmd and Nos2
Source: Nat Commun. 2023 Oct 21;14:6686. doi: 10.1038/s41467-023-42218-1 (PMC10590453; doi:10.1038/s41467-023-42218-1)
Supplement: Supplementary file 6 — Reporting Summary [file 41467_2023_42218_MOESM6_ESM.pdf]

nature portfolio

Corresponding author(s): Edward A. Miao

Last updated by author(s): Jun 7, 2023

Reporting Summary

Nature Portfolio wishes to improve the reproducibility of the work that we publish. This form provides structure for consistency and transparency in reporting. For further information on Nature Portfolio policies, see our Editorial Policies and the Editorial Policy Checklist.

Statistics

For all statistical analyses, confirm that the following items are present in the figure legend, table legend, main text, or Methods section.

n/a	Confirmed	
☐	☒	The exact sample size (*n*) for each experimental group/condition, given as a discrete number and unit of measurement
☐	☒	A statement on whether measurements were taken from distinct samples or whether the same sample was measured repeatedly
☐	☒	The statistical test(s) used AND whether they are one- or two-sided<br>*Only common tests should be described solely by name; describe more complex techniques in the Methods section.*
☒	☐	A description of all covariates tested
☐	☒	A description of any assumptions or corrections, such as tests of normality and adjustment for multiple comparisons
☐	☒	A full description of the statistical parameters including central tendency (e.g. means) or other basic estimates (e.g. regression coefficient) AND variation (e.g. standard deviation) or associated estimates of uncertainty (e.g. confidence intervals)
☐	☒	For null hypothesis testing, the test statistic (e.g. *F*, *t*, *r*) with confidence intervals, effect sizes, degrees of freedom and *P* value noted<br>*Give P values as exact values whenever suitable.*
☒	☐	For Bayesian analysis, information on the choice of priors and Markov chain Monte Carlo settings
☒	☐	For hierarchical and complex designs, identification of the appropriate level for tests and full reporting of outcomes
☒	☐	Estimates of effect sizes (e.g. Cohen's *d*, Pearson's *r*), indicating how they were calculated

Our web collection on statistics for biologists contains articles on many of the points above.

Software and code

Policy information about availability of computer code

Data collection	Microscopy: Keyence BZ-X800/BZX810<br>Flow Cytometry: BD LSRFortessa X-20 Cell Analyzer<br>qPCR: QuantStudio 3 Real-Time PCR System (Applied Biosystems, A28567)<br>Nitrate and Nitrite Assay: Cayman Chemical Cat. No. 780001<br>ELISA: BioTek Synergy H1 Gen5 ver 3.10<br>LDH: CytoTox 96, Promega, Cat. G1780<br>Spatial Transcriptomics: 10x Genomics Visium
Data analysis	Microscopy: Keyence BZ-X800/BZX810, Microsoft Windows 10 Paint v21H2, ImageJ win-64 v1.53<br>Flow Cytometry: FlowJo Windows v10.7.1<br>qPCR: ΔΔCt method, Microsoft excel 2013<br>Nitrate and Nitrite Assay: BioTek Synergy H1 Microplate Reader<br>ELISA:Gen5 ver 3.10 and Microsoft excel 2013<br>LDH: Microsoft excel 2013<br>Spatial Transcriptomics: Space Ranger, Loupe Browser, Seurat R package version 4 (including ggplot2 package), and Microsoft excel 2013<br>Statistical analyses: GraphPad Prism 9

For manuscripts utilizing custom algorithms or software that are central to the research but not yet described in published literature, software must be made available to editors and reviewers. We strongly encourage code deposition in a community repository (e.g. GitHub). See the Nature Portfolio guidelines for submitting code & software for further information.

Data

Policy information about availability of data

All manuscripts must include a data availability statement. This statement should provide the following information, where applicable:

- Accession codes, unique identifiers, or web links for publicly available datasets
- A description of any restrictions on data availability
- For clinical datasets or third party data, please ensure that the statement adheres to our policy

All relevant data are included in the Article or its Supplementary Information and Source Data files. More details are available from the corresponding authors upon request. The Mouse Cell Atlas data used for spatial genomics is previously published under MCA1.0 (DOI: https://doi.org/10.1016/j.cell.2018.02.001)

Research involving human participants, their data, or biological material

Policy information about studies with human participants or human data. See also policy information about sex, gender (identity/presentation), and sexual orientation and race, ethnicity and racism.

Reporting on sex and gender	N/A
Reporting on race, ethnicity, or other socially relevant groupings	N/A
Population characteristics	N/A
Recruitment	N/A
Ethics oversight	N/A

Note that full information on the approval of the study protocol must also be provided in the manuscript.

Field-specific reporting

Please select the one below that is the best fit for your research. If you are not sure, read the appropriate sections before making your selection.

☒ Life sciences ☐ Behavioural & social sciences ☐ Ecological, evolutionary & environmental sciences

For a reference copy of the document with all sections, see nature.com/documents/nr-reporting-summary-flat.pdf

Life sciences study design

All studies must disclose on these points even when the disclosure is negative.

Sample size	Target sample size was 6 mice per group based on power analysis and historical trends in data variance, however smaller or larger group sizes were used sometimes due to mouse availability. 6 mice are the preferred number to be used per group based on power analysis using the two tailed Student's t-test. The following parameters based on our prior experiments were used in power analysis (we use the website "Handbook of Biological Statistics, by John H. McDonald for reference). In a prior experiment that is typical of infection models in our lab, we find that the standard deviation for bacterial counts in wr infections is 1404 CFU (data is presented as log transformation; log(1404) = 0.17). We assume the standard deviation for the test infection will be similar. We wish to detect differences with a p value of 0.05 (called alpha in the power analysis). The power of the experiment is the percent chance to find the indicated difference if it exists. We wish to have an 80 percent chance to find a 2 fold difference (log10(2) = 0.301) and a 99% chance to detect a 3-fold difference in the CFU (log10(3) = 0.477). We will have an equal number of mice in each group (sample size ratio Nl /N2). Key values in historical data that were used for this power analysis are as follows CFU/mg of spleen from a Salmonella infection: 4079, 6168, 3545, 5371, 1823, ,4963; average 4325; stdev 1404 (we use the log transformed data for the power analysis which are 3.61, 3.79, 3.55, 3.73, 3.26, 3.70; average 3.61; with a standard deviation of 0.17). The desired minimum difference for a 2 fold effect is 8650 (log transformed 3.94). However, when mouse numbers are limited, sometimes we use fewer mice, accepting that we may not detect more subtle differences between groups. When higher mouse numbers are available, we use larger numbers of mice to ensure detection of statistically significant differences.
Data exclusions	In some experiments we excluded mice that died before tissue collection for histological scoring or CFU determination, as stated in main text and/or indicated in figure legends and graphical figure panels (e.g. Fig. 6C)
Replication	Every experiment was replicated at least in 2 separate cohorts, and in some up to 10 independent experiments. Experiments with one replication are presented and only in the Supplemental Figures and number of replicates are indicated.
Randomization	All samples were allocated into experimental groups in a non-biased manner. Covariant controls are not relevant because we use inbred mice in all experiments. For mice, sexes were evenly distributed between groups.
Blinding	Experimental group assignments were not blinded because all mice are genetically identical (except for X and Y chromosomes, which were distributed equally between groups). All histological pathology scoring were blinded.

Reporting for specific materials, systems and methods

We require information from authors about some types of materials, experimental systems and methods used in many studies. Here, indicate whether each material, system or method listed is relevant to your study. If you are not sure if a list item applies to your research, read the appropriate section before selecting a response.

Materials & experimental systems

n/a	Involved in the study	
☐	☒	Antibodies
☐	☒	Eukaryotic cell lines
☒	☐	Palaeontology and archaeology
☐	☒	Animals and other organisms
☒	☐	Clinical data
☒	☐	Dual use research of concern
☒	☐	Plants

Methods

n/a	Involved in the study	
☒	☐	ChIP-seq
☐	☒	Flow cytometry
☒	☐	MRI-based neuroimaging

Antibodies

Antibodies used	Primary antibodies IHC: C. violaceum, 1:2000 (rabbit antisera custom generated by Cocalico Biologicals); Ly6G, 1:300 (Biolegend Cat. No. 127601); CD68, 1:200 (Abcam Cat. No. ab125212); CD3, 1:500 (Abcam Cat. No. ab5690); GFP, 1:200 (Invitrogen Cat. No. A11122); secondary antibody polymer (Cell Signaling-Signal Stain boost, HRP anti-rabbit, 8114; ImmPRESS® HRP Goat Anti-Rat IgG Polymer Detection Kit, Peroxidase, Cat. No. MP-7404-50). Primary antibodies IF: Ly6G Alexa Fluor 647, 1:100 (Biolegend Cat. No. 127610), CD68 Alexa Fluor 488, 1:100 (Abcam Cat. No. ab201844), iNOS Alexa Fluor 568, 1:100 (Abcam Cat. No. ab209595), e-cadherin, 1:200 (Abcam Cat. No. ab15148); secondary antibody (Invitrogen Goat-anti-rabbit 594, 1:1000, Cat. No. A32740). Flow Cytometry antibodies: Live-or-Dye™ fixable viability dye in APC-Cy™7 (Biotium, Cat. No. 32008, according to product manual), Mouse BD Fc Block™ (BD Biosciences, Cat. No. 553142, according to product manual) rat anti-mouse Ly-6G in BV421™ at 1:300 for 30 minutes (BD Horizon™, Cat. No. 562737), and finally, rat anti-mouse CD68 in FITC at 1:300 for 30 minutes (BioLegend®, Cat. No. 137005). Antibodies for neutrophil depletion: anti-mouse Ly6G/Ly6C, 0.2mg/mouse (BioXcel Cat. No. BE0075); isotype control anti-keyhole limpet hemocyanin, 0.2mg/mouse (BioXcel Cat. No. BE0090).
Validation	Anti-Chromobacterium violaceum antibody was is validated in house by staining only to bacteria sized and shaped objects, and not staining outside the infected lesions (lesions were identified by Hematoxylin or DAPI) and by no staining from pre-bleed serum from host animal before C. violaceum infection. Uninfected mouse livers were also stained with anti-C. violaceum antibody to confirm no unspecific binding. For IHC primary antibodies we validated using a control tissue (spleen) to confirm CD68, CD3, and NCR1-GFP; Ly6G was confirmed by staining blood from mice. For IHC secondary antibody polymer, infected mice livers were stained with secondary antibody only to validate no background from secondary. For IF primary antibodies we validated using control tissue (spleen) and comparison of granuloma architecture to IHC stained tissues (Ly6G and CD68). Primary anti-iNOS antibody was validated in Nos2 KO mice which do not produce iNOS. For IF secondary antibodies, infected mice livers were stained with secondary antibody only to validate no background from secondary. Flow antibodies were chosen based on validated IHC antibodies (Ly6G and CD68) along with unstained and single stained controls. All antibody staining was visually validated by board-certified animal pathologist.

Eukaryotic cell lines

Policy information about cell lines and Sex and Gender in Research

Cell line source(s)	Primary mouse bone marrow derived macrophages.
Authentication	Macrophages differentiated from bone marrow using L-Cell media; authenticated via morphology but no other authentication.
Mycoplasma contamination	The cells were tested negative for mycoplasma.
Commonly misidentified lines (See ICLAC register)	No commonly misidentified cell lines were used.

Animals and other research organisms

Policy information about studies involving animals; ARRIVE guidelines recommended for reporting animal research, and Sex and Gender in Research

Laboratory animals	Previously generated mouse lines were used in this study: wild type (WT) C57BL/6, Prf1 null mice, Ncf1 null mice, Casp7 null mice, Nos2 null mice, Gsdmd null mice, Casp1 null Casp11 null mice, Acod1 null mice, Rag1 null mice. All strains were maintained in C57BL/6 background. For mouse infections, 8-12-week-old mice were used. All strains were maintained on 12/12 light cycles, at 72 +/- 2 °F, and under the humidity set point of 45%.
Wild animals	No wild animals were used.

Reporting on sex	Male and female mice were used in equivalent numbers unless otherwise indicated.
Field-collected samples	No field-collected samples were used.
Ethics oversight	Animal protocols were approved by the Institutional Animal Care and Use Committee at the University of North Carolina at Chapel Hill, or by the Institutional Animal Care and Use Committee at Duke University and met guidelines of the US National Institutes of Health for the humane care of animals.

Note that full information on the approval of the study protocol must also be provided in the manuscript.

Flow Cytometry

Plots

Confirm that:

☒ The axis labels state the marker and fluorochrome used (e.g. CD4-FITC).

☒ The axis scales are clearly visible. Include numbers along axes only for bottom left plot of group (a 'group' is an analysis of identical markers).

☒ All plots are contour plots with outliers or pseudocolor plots.

☒ A numerical value for number of cells or percentage (with statistics) is provided.

Methodology

Sample preparation	Whole livers were harvested at the indicated time points post infection. Briefly, mice were euthanized according to IACUC guidelines, followed by whole body perfusion with PBS (Gibco, Cat. No. 14190-144). Whole livers were harvested and minced on ice using scissors, followed by incubation in digestion buffer (100U/mL Collagenase Type IV (Gibco, Cat. No. 17104019) prepared in DMEM (Gibco, Cat. No. 11885-084), supplemented with 1 mM CaCl2 and 1 mM MgCl2,) at 37°C for 40 minutes with intermittent vortexing. Digested tissues were mechanically homogenized through a Falcon® 40 µm cell strainer (Corning, Cat. No. 352340) to remove the majority of hepatocytes, and washed twice with RPMI (Gibco™, Cat. No. 11875-093) supplemented with 1% FBS (CPS Serum, Cat. No. FBS-500-HI) and 1X penicillin/streptomycin (Gibco, Cat. No. 15140-122), followed by centrifugation in an Eppendorf® centrifuge (model 5810 R) at 1200 rpm (290g) for 8 minutes at room temperature. Leukocytes were further isolated using a Percoll® gradient: samples were resuspended in 45% Percoll® (GE Healthcare, Cat. No. 17-0891-01), prepared in DMEM + 1.5M NaCl), with an 80% Percoll® (prepared in PBS + 1.5 M NaCl) underlay, and spun for 20 minutes at 2000 rpm (805g), room temperature, with no brake. Following collection of the leukocyte layer at the gradient interface, samples were washed twice, as before, and red blood cells were lysed with 1X RBC Lysis Buffer (eBioscience, Cat. No. 00-4333-57, according to product manual). Cells were washed and counted using trypan blue. 1x106 cells from each liver were stained for various cell markers: Live-or-Dye™ fixable viability dye in APC-Cy™7 (Biotium, Cat. No. 32008, according to product manual), Mouse BD Fc Block™ (BD Biosciences, Cat. No. 553142, according to product manual) rat anti-mouse Ly-6G in BV421™ at 1:300 for 30 minutes (BD Horizon™, Cat. No. 562737), and finally, rat anti-mouse CD68 in FITC at 1:300 for 30 minutes (BioLegend®, Cat. No. 137005) using Intracellular Fixation & Permeabilization Buffer (eBioscience, Cat. No. 88-8824-00, according to product manual).
Instrument	BD LSRFortessa X-20 Cell Analyzer.
Software	FlowJo (for Windows, version 10.7.1)
Cell population abundance	Cell populations varied depending on the mouse genotype. For infected WT livers, macrophages made up approx. 50% of single cells and neutrophils made up approx. 15% of single cells.
Gating strategy	An example of the gating strategy is present in Supplementary Figure 5d. Total leukocytes were gated using FSC/SSC. From there, FSC-H/FSC-A was used to gate on single cells. Finally, macrophages and neutrophils were distinguished with CD68 and Ly6G, respectively.

☒ Tick this box to confirm that a figure exemplifying the gating strategy is provided in the Supplementary Information.
